# Supplementary material for: Mobilization of cholesterol induces the transition from quiescence to growth in Caenorhabditis elegans through steroid hormone and mTOR signaling
Source: Commun Biol. 2024 Jan 24;7:121. doi: 10.1038/s42003-024-05804-7 (PMC10808130; doi:10.1038/s42003-024-05804-7)
Supplement: Supplementary file 2 — Supplementary information [file 42003_2024_5804_MOESM2_ESM.pdf]

Supplementary material for

Mobilization of cholesterol induces the transition from quiescence to growth in *Caenorhabditis elegans* through steroid hormone and mTOR signaling

Kathrin Schmeisser[1,4], Damla Kaptan[1], Bharath Kumar Raghuraman[1], Andrej Shevchenko[1], Jonathan Rodenfels[1,2], Sider Penkov[1,3], Teymuras V. Kurzchalia[1,4]

1. Max Planck Institute of Molecular Cell Biology and Genetics, Dresden, Germany
2. Physics of Life (PoL), Technical University Dresden, Dresden, Germany
3. Center of Membrane Biochemistry and Lipid Research, Faculty of Medicine, Technical University Dresden, Dresden, Germany
4. Corresponding authors: Teymuras V. Kurzchalia (t.kurzchalia@gmail.com); Kathrin Schmeisser (kathrin.schmeisser@mpi-cbg.de)

Supplementary figure 1:

Characterization of SCL-12 expression during dauer entry and exit

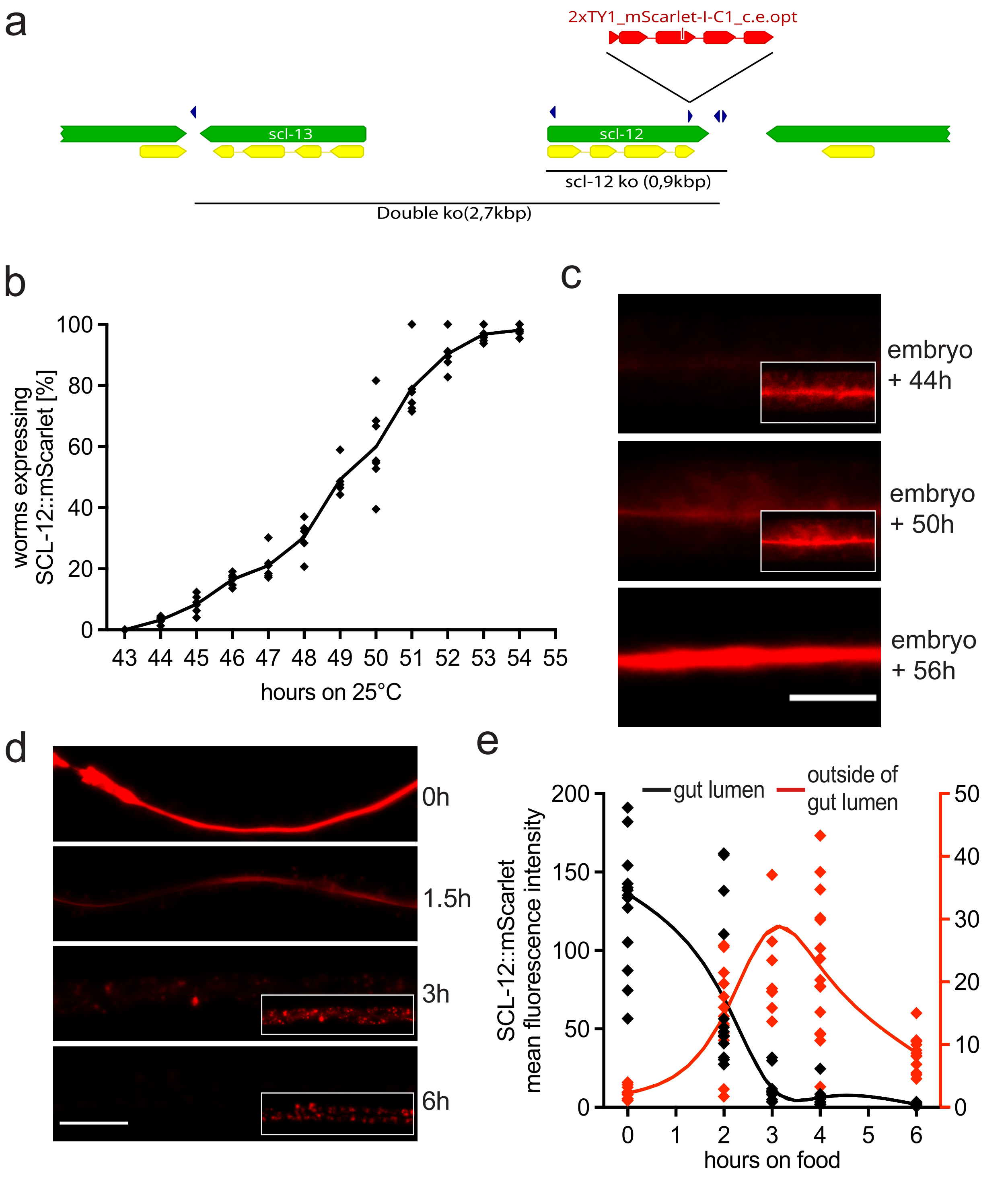

Supplementary figure 2:
Characterization of scl-12&13 mutants in dauer state

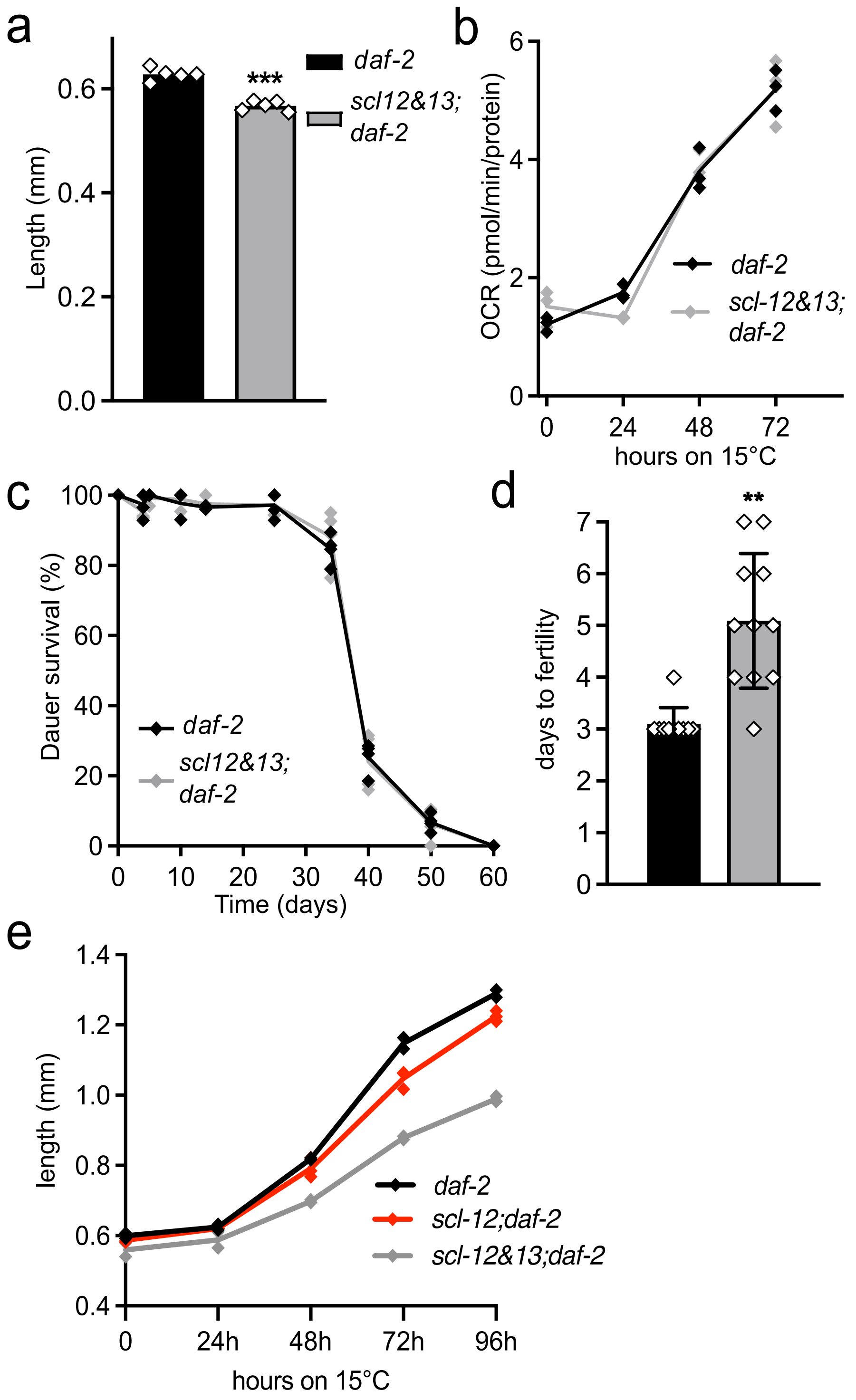

Supplementary figure 3:

Dauer exit depends on intact lysosomes

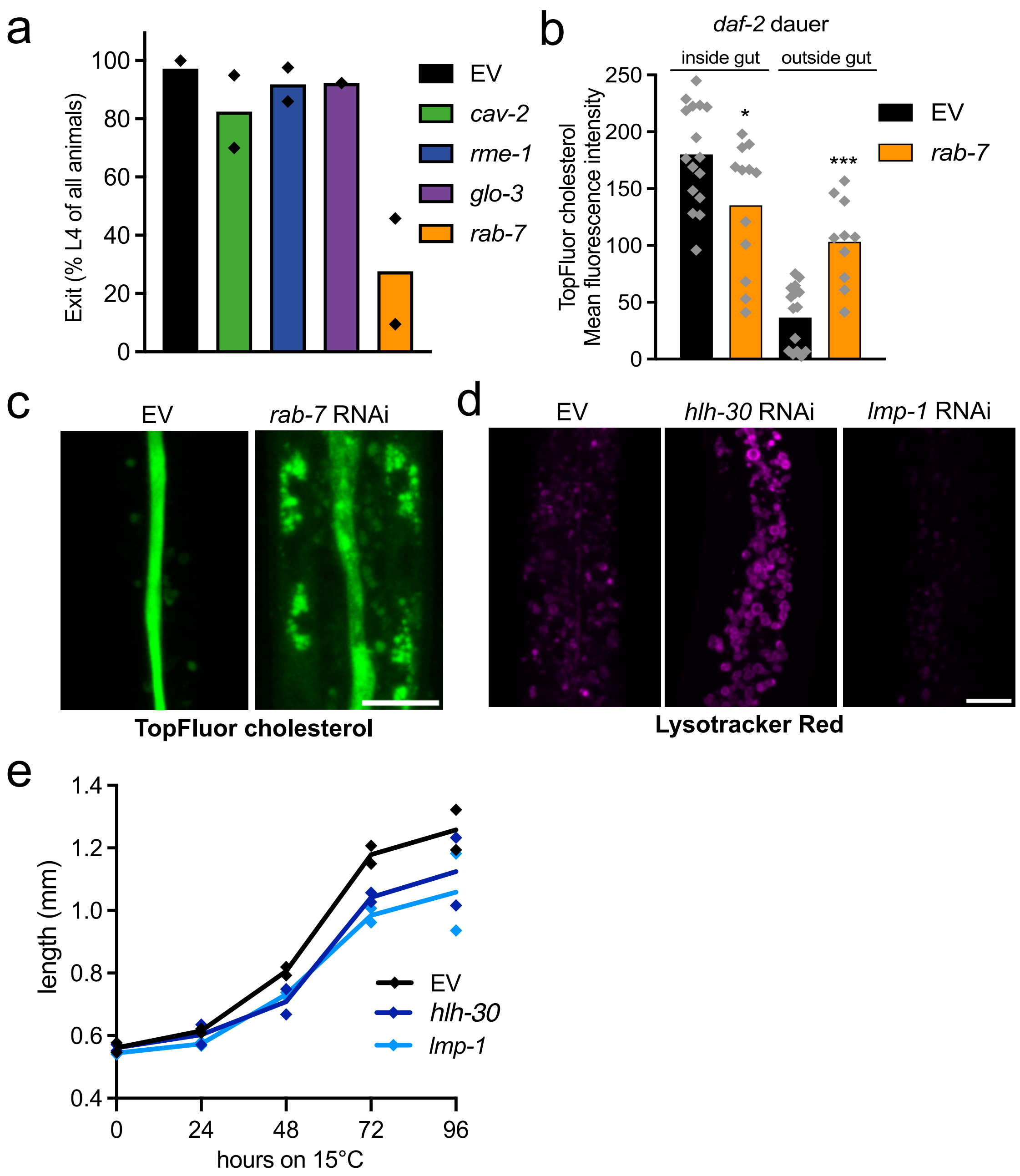

Supplementary figure 4:
Characterization of daf-15::mNeonGreen::AID;TIR1 animals

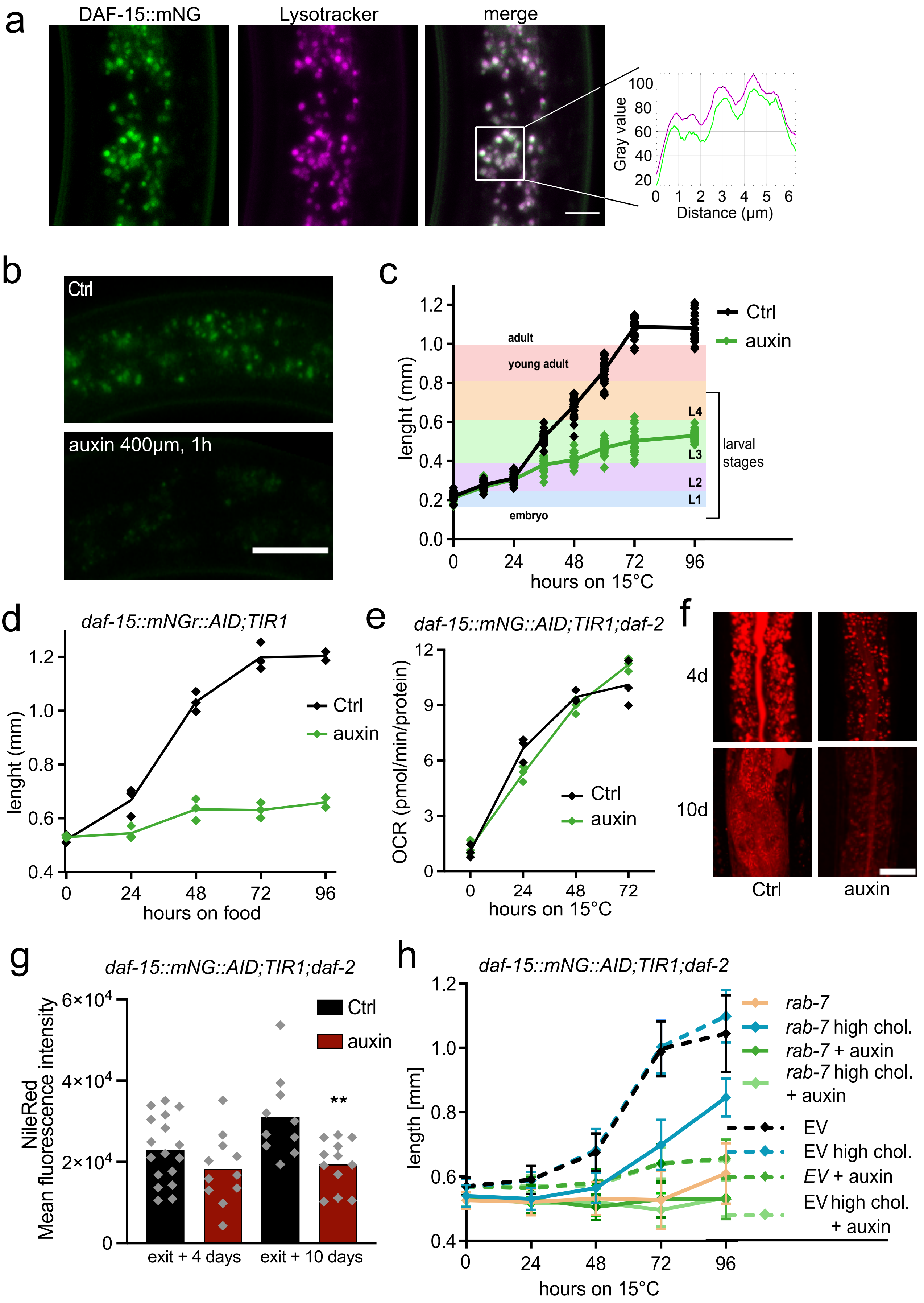

Supplementary figure 5:
Orthologues of SLC38A9 and NPC1 do not affect dauer exit

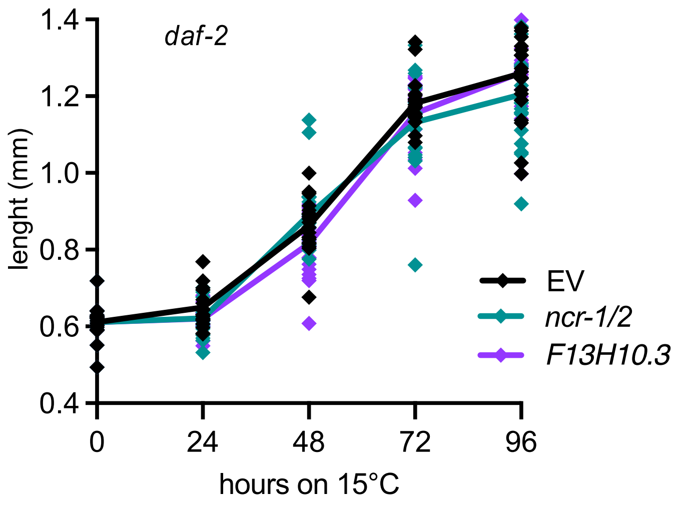

Supplementary figure legends

Figure S1: Characterization of SCL-12 expression during dauer entry and exit

a Genomic engineering of *scl-12&13* and *scl-12* knock-out mutants and SCL-12 reporter animals. **b** SCL-12::mScarlet;*daf-2* reporter animals expressing SCL-12::mScarlet as indicated by appearance of fluorescence after synchronization in % per population. **c** Representative fluorescent micrographs of SCL-12::mScarlet;*daf-2* reporter animals while entering dauer at 44 h, 50 h and 56 h after synchronization of eggs. 44 h and 50 h insets are brightness adjusted images for increased visibility. Scale bar: 15 µm. **d** SCL-12::mScarlet reporter starvation dauers (wt) while exiting dauer at 1.5 h, 3 h and 6 h after introduction of food. 3 h and 6 h insets are brightness adjusted images. Scale bar: 25 µm. **e** Mean fluorescence intensity of SCL-12::mScarlet reporter starvation dauers (0 h) and while exiting dauer via introduction of food. Black: Fluorescence in the gut lumen. Red: Fluorescence outside of the lumen. Means and individual values of at least 10 individual worms per time point.

Figure S2: Characterization of *scl-12&13* mutants in dauer state

a Lenght in mm of *daf-2* (black) and *scl-12&13;daf-2* (gray) dauer larvae. Average and individual values of 5 independent experiments with at least 20 worms per strain, unpaired two-tailed t-test showed $p < 0.0001$. **b** Oxygen consumption rate (OCR) in pM (picomolar)/min/µg protein of *daf-2* and *scl-12&13;daf-2* dauers as worms exit the dauer state. Average and individual values of 3 measurements of 6 technical replicates in one representative experiment. Two-way RM ANOVA with Geisser-Greenhouse correction comparing the strains showed $p = 0.8572$. **c** Dauer survival. Average and individual values of one experiment with 3 - 4 biological replicates. **d** Time to reach fertility as indicated by egg laying after dauer exit induced by temperature switch to 15°C. Average ± SD and individual values of 10 worms per strain, paired two-tailed t-test showed $p = 0.0034$. **e** Growth/length in mm of *daf-2*, single mutants *scl-12;daf-2*, and double mutants *scl-12&13;daf-2* after induction of dauer exit by temperature switch to 15°C. Average and individual values of 2 independent experiments with a minimum of 10 worms per condition, two-way RM ANOVA with Geisser-Greenhouse correction comparing *daf-2* versus *scl-12;daf-2* showed $p = 0.0364$, and *daf-2* versus *scl-12&13;daf-2* $p = 0.0053$.

Figure S3: Dauer exit depends on intact lysosomes

a Percentage of *daf-2* populations that develop into L4 larvae 48 h after dauer recovery was induced by temperature switch to 15°C on RNAis against *cav-2* (green), *rme-1* (blue), *glo-3* (purple), and *rab-7* (orange), compared to EV (black). Average and individual values of 2

experiments (1 experiment for *glo-3* RNAi) with a minimum of 15 individual worms per condition. **b** *daf-2* dauers fed with *rab-7* and EV RNAi and TopFluor cholesterol, mean fluorescence intensity inside and outside of the gut. Mean and individual values of a minimum of 12 animals per condition, unpaired t-test comparing EV and *rab-7* RNAi inside the gut showed p = 0.0256 and outside of the gut p < 0.0001. **c** Representative images of **b**. **d** Representative images of *daf-2* dauers treated with RNAis against *hlh-30* and *lmp-1*, compared to EV, labelled with Lysotracker dye (magenta). **e** Growth/length in mm in *daf-2* exiting dauers induced by temperature switch to 15°C on RNAis against *hlh-30* (dark blue) and *lmp-1* (light blue), compared to EV (black). Means and individual values of 2 experiment with a minimum of 15 worms per condition, two-way ANOVA test comparing EV versus *hlh-30* showed p < 0.023 and EV versus *lmp-1* p = 0.0047.

Figure S4: Characterization of *daf-15::mNeonGreen::AID;TIR1* animals

a DAF-15::mNeonGreen;*daf-2* reporter dauers (DAF-15::mNG; green) labelled with Lysotracker dye (magenta). The similar profile of both fluorophores indicates co-localization. Scale bar: 5 µm. **b** *daf-15::mNeonGreen::AID;TIR1* dauers treated with 400 µM auxin for 1 h compared to control (ethanol). **c** Growth in mm of *daf-15::mNeonGreen::AID;TIR1* worms synchronized as L1 on auxin (400 µM; green) compared to the solvent control (black). Sizes of developmental stages are indicated in the graph. Average and individual values of one experiment with 30 worms per condition, two-way RM ANOVA with Geisser-Greenhouse correction showed p < 0.0001. **d** Growth in mm in *daf-15::mNeonGreen::AID;TIR1* exiting starvation dauers treated with auxin (green) or solvent control (black). Average and individual values of 3 independent experiments (96 h: 2 experiments) with 30 worms per condition, two-way RM ANOVA with Geisser-Greenhouse correction showed p < 0.0001. **e** OCR in pM/min/µg protein in *daf-15::mNeonGreen::AID;TIR1;daf-2* during dauer exit induced by temperature switch, on auxin or solvent control. Average and individual values of 3 measurements of 6 technical replicates in one representative experiment. Two-way RM ANOVA with Geisser-Greenhouse correction comparing Ctrl and auxin treatment showed p = 0.4853. **f** Representative images of G. **g** Nile Red lipid staining in *daf-15::mNeonGreen::AID;TIR1;daf-*2 exiting dauers treated with auxin (red) and the respective control (black) at day 4 and 10 after dauer induction via temperature shift. Average and individual values of at least 10 worms per condition, an unpaired two-tailed t test showed p = 0.158 for Ctrl versus auxin at day 4 of exit and p = 0.0034 at day 10 of exit. **h** Extended version of fig. 6e including EV control: Growth/length in mm in *daf-15::mNeonGreen::AID;TIR1;daf-2* exiting dauers on RNAi against *rab-7* compared to EV (dotted lines), treated with 1 mM cholesterol (turquoise) or 13 µM cholesterol (black/orange) and with (green) or without 400 µM auxin (light green). Average ± SD of one experiment with a minimum of 25 individual

worms per condition, two-way ANOVA with post hoc Tukey's test comparing *rab-7* with *rab-7* high chol. showed $p < 0.0001$, *rab-7* HC with *rab-7* high chol. + auxin $p < 0.0001$, EV with EV + auxin $p < 0.0001$, EV with EV high chol. + auxin $p < 0.0001$, EV high chol. with EV high chol. + auxin $p < 0.0001$. Individual values can be found in the raw data file 6e_S4h.

Figure S5: Orthologues of SLC38A9 and NPC1 do not affect dauer exit

Growth/length in mm in *daf-2* exiting dauers induced by temperature switch to 15°C on RNAis against an OD-adjusted mix of *ncr-1* and *ncr-2* (teal) and *F13H10.3* (purple), compared to EV (black). Means and individual values of 1 experiment with a minimum of 15 worms per condition, two-way ANOVA test comparing EV versus *ncr-1;ncr-2* showed $p = 0.1146$ and EV versus *F13H10.3* $p = 0.0653$.
